# Effects of Planting Methods and Varieties on Rice Quality in Northern China

**DOI:** 10.3390/foods14071093

**Published:** 2025-03-21

**Authors:** Lili Wang, Liying Zhang, Na He, Changhua Wang, Yuanlei Zhang, Zuobin Ma, Wenjing Zheng, Dianrong Ma, Hui Wang, Zhiqiang Tang

**Affiliations:** 1 Rice Research Institute of Liaoning Province, Liaoning Academy of Agricultural Sciences, Shenyang 110101, China; wll1230510@163.com (L.W.);; 2 Liaoning Academy of Agricultural Sciences, Shenyang 110101, China

**Keywords:** planting method, variety, rice quality

## Abstract

With the continuous improvement in living standards, consumers’ demand for rice quality has been increasingly growing. This study analyzed the quality characteristics of different rice varieties under various cultivation methods. This study examined the rice variety Liaoxing 21 (LX21), the upland rice variety Han 9710 (H9710), and the hybrid rice variety Liaoyou 7362 (LY7362) from Liaoning Province to evaluate the effects of transplanting (TP) and direct seeding (DS) on processing, appearance, nutritional, and tasting quality. The results indicated that the planting method (PM) had a relatively minor impact on processing quality. Compared to TP, DS significantly increased grain length (GL) by 1.19%, grain width (GW) by 2.69%, appearance (A) by 2.61%, stickiness (Ss) by 7.15%, degree of balance (DB) by 3.19%, apparent amylose content (AAC%) by 6.20%, fa by 0.66%, fa/fb_3_ by 5.34%, and protein content (PC) by 19.93%. However, DS significantly reduced the grain length/width ratio (GL/W) by 1.03%, chalky grain rate (CGR) by 46.00%, chalkiness (CH) by 52.76%, and fb_3_ by 4.23%. Compared to DS, TP resulted in a higher peak viscosity (PV), final viscosity (FV), and pasting temperature (PaT), whereas setback (SB) was lower. Among the tested varieties, LX21 exhibited superior milled rice rate (MRR), head rice rate (HRR), GL, GL/W, A, Ss, DB, taste value (T), and FV compared to H9710 and LY7362, while demonstrating significantly lower CGR, CH, hardness (H), fa, trough viscosity (TV), and peak time (PeT). Under the same planting conditions, the conventional rice variety LX21 demonstrated excellent processing, appearance, and taste quality, whereas H9710 exhibited favorable nutritional quality and Rapid Visco Analyzer (RVA) characteristics. Meanwhile, we also analyzed the correlation between temperature/light conditions and nutritional quality, as well as RVA profiles. The results showed that variations in temperature and light were closely associated with amylopectin accumulation and starch pasting properties. This study highlights the findings that selecting the appropriate PMs and japonica rice varieties can effectively enhance overall rice quality. In the medium maturing regions of Liaoning Province, adopting DS with medium–early maturing japonica rice varieties offers an optimal production strategy for achieving high quality, high yield, and efficient utilization of temperature and light resources.

## 1. Introduction

As one of the most important food crops, rice provides 35–60% of dietary energy for over 3 billion people globally. In China, it is the primary food source for approximately 60% of the population. Therefore, exploring the impact of rice cultivation methods on yield and quality has significant academic and practical value [1,2]. Rice yield and quality can be influenced by various factors, including breeding variety, cultivation environment, fertilization management, water management, and pest and disease control. Optimizing these factors can effectively enhance the yield and nutritional value, thereby addressing the food demands of a growing population. Traditional manual transplanting (TP) is no longer the only planting method, as rice cultivation now includes several techniques such as seedling throwing, mechanical transplanting, and direct seeding (DS), with TP and DS being the most widely used [3]. TP improves seed germination and seedling establishment rates through pre-transplant seedling management, whereas appropriate row and plant spacing after transplantation enhances agricultural management and yield [4]. In contrast, DS significantly reduces production costs by lowering labor and fertilizer inputs, eliminating the need for seedling fields, shortening the growth cycle in the main field, and improving mechanization levels [5].

Rice quality is primarily evaluated based on processing, appearance, eating, and nutritional qualities [6,7]. Processing quality refers to the entire process, from the paddy harvest to the final product reaching consumers, including husking, bran removal, and polishing. The key indicators affecting processing quality include the brown rice rate (BRR), milled rice rate (MRR), and head rice rate (HRR) [8]. Appearance quality is the most direct indicator of rice quality and plays a crucial role in determining its market price and usage [9,10]. Eating quality is a fundamental aspect of rice quality and a key determinant of its market value. Because starch constitutes the largest proportion of rice, its composition and structure significantly influence cooking and eating quality, with apparent amylose content (AAC%) being the most critical factor [11]. Rice serves as a staple food in the human diet, providing not only abundant carbohydrates but also essential nutrients such as proteins, fats, amino acids, and minerals. Rice proteins contribute to approximately 15% of the global edible protein supply, rendering rice a vital source of dietary protein [12]. Additionally, the Rapid Visco Analyzer (RVA) is widely used to assess the viscosity characteristics of milled rice, providing results that are closely associated with eating quality traits [13].

This study adopted two planting methods (PMs), TP and DS, to investigate the quality-related traits of three rice varieties. Liaoxing 21 (LX21) is a japonica conventional rice cultivar, cultivated in mid-early maturing rice regions of eastern and northern Liaoning Province; Han 9710 (H9710) is a japonica conventional upland rice cultivar, planted in central and northern Liaoning, Jilin Province, and southern Inner Mongolia; and Liaoyou 7362 (LY7362) is a japonica three-line hybrid rice cultivar, grown in mid-late maturing rice regions south of Shenyang. All these varieties have been officially approved and are widely promoted and cultivated. The above-mentioned cultivars belong to different types of japonica rice, and have all been officially approved and widely promoted and cultivated. The analysis of the quality of these three rice cultivars can provide theoretical value for the analysis of different types of rice quality in northern regions. A comparative analysis was conducted on the processing quality, appearance quality, eating and nutritional characteristics, and viscosity characteristics of the RVA profile along with a correlation analysis between the nutritional quality of cooked rice and RVA values. Analysis of the correlation between temperature/light conditions, nutritional quality, and RVA profiles revealed that variations in temperature and light were closely associated with amylopectin accumulation and starch pasting properties. This study filled a research gap in understanding the impact of single-season rice cultivation patterns in Northeast China on the rice quality of different rice varieties, which can provide a reference for more rice farmers to choose cultivation methods and rice varieties.

This study investigated the effects of planting methods and different rice varieties on rice processing, appearance, nutritional, tasting, and RVA quality. The specific objectives were as follows: (1) to evaluate the impacts of planting methods and varieties on rice quality; (2) to determine the optimal combinations of planting methods and rice varieties; and (3) to analyze the relationships among rice nutritional quality, RVA profiles, and temperature/light characteristics.

## 2. Materials and Methods

### 2.1. Experimental Materials

The experiment was conducted at the experimental base of the Liaoning Rice Research Institute (41°46′ N, 123°35′ E) on medium-fertility clay loam soil. The basic physical and chemical properties of the plow layer were as follows: available nitrogen content of 107.2 mg/kg, available phosphorus content of 23.5 mg/kg, available potassium content of 145.6 mg/kg, organic matter content of 18.7 g/kg, and a pH value of 5.4. The tested materials included conventional japonica rice LX21, hybrid rice LY7362, and dry-direct-seeding variety H9710.

### 2.2. Experimental Design

This experiment employed a split-plot design, with PMs as the main plots and varieties as subplots. To ensure effective isolation between fields using different PMs, a plastic film was applied to wrap the ridges.

DS: Seeding was conducted on April 26 using mechanized dry drilling, with a seeding rate of 5.5 million seeds per hectare and a row spacing of 30 cm.

TP: Seeding was conducted on April 16, followed by transplanting on May 21, when the seedlings had reached a leaf age of 3.5 leaves. The TP row-to-plant spacing was 30 cm × 13.3 cm, with 3–4 seedlings per hill. Each plot covered an area of 120 m^2^ with three replicates. The main growth stages of the rice varieties under different PMs are presented in Table 1.

The total nitrogen application rate in this experiment was 225 kg/ha, with a base, tiller, and panicle fertilizer application ratio of 4:3:3. Under the TP, tiller fertilizer was applied once when the leaf age index reached 60%, and panicle fertilizer was applied once at 80%. In the DS, tiller fertilizer was applied at the 3-leaf-1-heart stage, while panicle fertilizer was adopted once at an 80% leaf-age index. The nutrient application ratio of N:P_2_O_5_: K_2_O was 2:1:1, with phosphate fertilizer applied entirely as a base fertilizer and potash fertilizer applied in two equal doses as the base fertilizer and again at the jointing stage.

### 2.3. Methods for Measuring Indicators

#### 2.3.1. Main Quality Indicators of Rice

After maturation, the tested materials were harvested, air-dried, and stored at room temperature for three months to balance the moisture content before being used for rice quality determination. Prior to analysis, all samples were uniformly winnowed using an winnowing machine(Hebei Nongfeng Machinery Co., Ltd., 42X-750, Shijiazhuang, China.). The determination methods for quality traits, including BRR, MRR, HRR, chalky grain rate (CGR), and grain length (GL), followed the National Standard of the People’s Republic of China “GB/T 1789-1999 High-Quality Paddy” [14]. Rice was ground into flour using a grinder (Beijing Guangming Medical Instrument Co., Ltd., Beijing, China, High-Speed Universal Grinder, Model FW-80) and sieved through a 100-mesh sieve to obtain refined rice flour for quality trait analysis. The protein content (PC%) in refined rice was determined by measuring the total nitrogen content in the rice flour using a Kjeldahl nitrogen analyzer (Kjeltec 8400, FOSS, Hillerød, Denmark) and calculated by multiplying the nitrogen content by the conversion factor of 5.95. The AAC% in rice flour was determined following the NY147-88 standard issued by the Ministry of Agriculture [15], using four reference samples (AC: 1.5%, 10.6%, 16.4%, and 25.6%) purchased from the China National Rice Research Institute.

In this study, Fa and Fb_3_ contents were determined following the methods described by [16,17]. A total of 0.1 g of deproteinized rice flour was dispersed in 1 mL of 95% ethanol solution, followed by the addition of 9 mL of 1 mol/L sodium hydroxide solution. The mixture was maintained at 5°C for 1 h for alkali gelatinization, heated in boiling water for 10 min, and subsequently cooled to room temperature. The gelatinized solution was diluted to a fixed volume, and 5 mL was transferred to another volumetric flask, where 1 mL of 1 mol/L acetic acid and 2 mL of KI-I_2_ solution were added before further dilution to a fixed volume. A spectrophotometer was used to scan the wavelengths from 200 to 900 nm to determine the peak wavelength of starch–iodine adsorption (λ_max_) and its corresponding absorbance (A_λmax_). Parameter F_2_ represents the percentage of the area between 400 nm and λ_max_ within the total area from 200 to 900 nm, and the mass fractions of Fa and Fb_3_ were calculated using Equations (1)–(3).

(1) $$\lambda_{max}^{\prime }=\frac{73.307\times A_{\lambda_{max}}+0.111\lambda_{max}-73.016}{The\;\lambda_{max}\;of\;the\;test\;sample-The\;\lambda_{max}\;of\;glutinous\;rice}\;,$$

Fa% = −11.59 × F2 − 10.92 × λ_max_ + 34.429, (2)

(3) $${\mathrm{Fb}}_{3}\%=44.691\;\times \;A_{\lambda_{max}}-0.774,$$

Once the mass fractions of Fa and Fb_3_ were determined using this method, the value of Fb_1_ + Fb_2_ was calculated as Fb_1_ + Fb_2_ = 1 − (Fa + Fb_3_) and abbreviated as Fb_1 + 2_, representing the number of medium-length chains in the amylopectin side chains. Additionally, the ratio of short chains to long chains was expressed as Fa/Fb_3_.

#### 2.3.2. Determination of the Eating Quality Value of Cooked Rice

A total of 30.0 g of milled rice was weighed and soaked in distilled water at a rice-to-water ratio of 1:1.25 for 30 min at room temperature. Using the Japanese variety Koshihikari as the standard sample, comprehensive eating quality, hardness, and viscosity values were determined using a cooked rice eating quality analyzer (STA-1A, Satake Corporation, Moriguchi, Japan).

#### 2.3.3. Determination of the Viscosity of the RVA Profile of Rice

Starch viscosity characteristics were measured and analyzed using an RVA produced by Newport Scientific Instruments, Warriewood, NSW, Australia, along with the supporting software Thermal Cycle for Windows (TCW). The determination followed the procedures of the American Association of Cereal Chemists (AACC) [18]. For analysis, 3 g of refined rice flour was mixed with 25 mL of distilled water. The characteristic values of the RVA profile were primarily represented by peak viscosity (PV), trough viscosity (TV), final viscosity (FV), breakdown (BD; PV, hot paste viscosity), setback (SB; FV, PV), peak time (PeT), and pasting temperature (PaT), with the viscosity measured in centipoises (CP).

#### 2.3.4. Temperature- and Light-Related Data

Climatic data during the grain-filling period were obtained from meteorological stations in the city where the experimental base was located.

#### 2.3.5. Data Processing and Statistics

Data analyses were conducted using Data Processing System (DPS) version 14.10 (Hangzhou, China), professional statistical software26 [19] and Principal Component Analysis (PCA, Origin Lab 2021, Hampton, MA, USA).

## 3. Results and Analysis

### 3.1. Processing Quality of Rice

The processing quality of the tested varieties in this experiment was not significantly affected by the PM. Among the three varieties, no significant differences were observed in BRR. However, MRR and HRR demonstrated significant differences (Table 2). Compared with LY7362, LX21 exhibited a significant increase in MRR and HRR of 4.41% and 12.75%, respectively, indicating that the conventional variety LX21 had superior MRR and HRR compared with the hybrid rice variety LY7362.

### 3.2. Appearance Quality of Rice

The analysis of variance revealed significant differences in grain length (GL), grain width (GW), grain length/width ratio (GL/W), chalky grain rate (CGR), and chalkiness (CH) among different PM s and varieties, as well as under the interaction of PM × variety (Table 3). Compared with TP, DS significantly increased GL and GW by 1.19% and 2.69%, respectively, and significantly decreased GL/W, CGR, and CH by 1.03%, 46.00%, and 52.76%, respectively. LX21 exhibited significantly higher GL and GL/W values than H9710 and LY7362, whereas CGR and CH exhibited the opposite trend. Additionally, the upland variety H9710 exhibited a significant increase in GW compared with LX21 and LY7362. These results indicated that DS enhanced GL and GW while reducing CGR and CH, thereby improving the rice appearance quality. Furthermore, LX21 had a relatively low CGR and CH, making its overall appearance superior to those of H9710 and LY7362.

**Figure 1:**
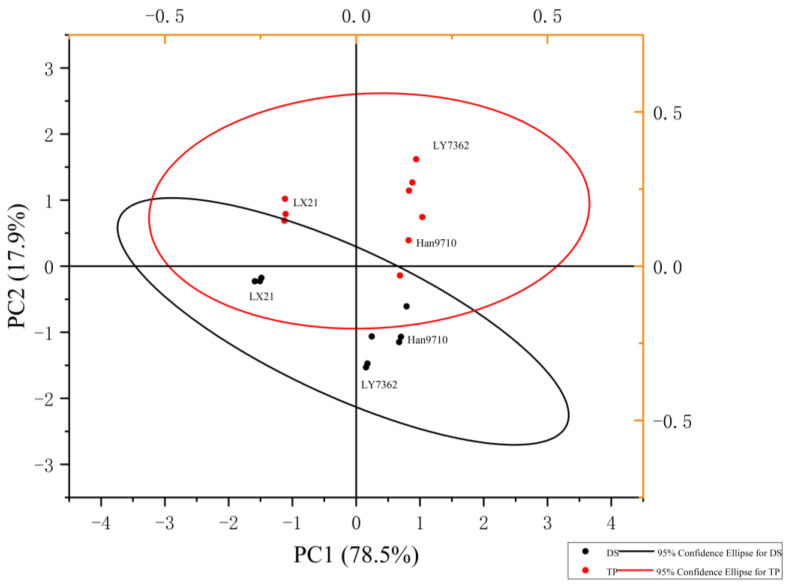
Correlation between planting method and appearance quality of rice. Note: TP is short for transplanting; DS is short for direct seeding; LX21 is short for Liaoxing21; H9710 is short for Han 9710; LY7362 is short for Liaoyou 7362.

Apparent differences in rice appearance quality were evident among these different treatment conditions, with significant differences between the rice appearance quality associated with TP and DS. Moreover, within each TP and DS, the rice appearance quality was distinctly separated from one another (Figure 1).

### 3.3. Tasting Quality of Rice

Significant differences were observed in appearance (A), hardness (H), stickiness (Ss), degree of balance (DB), and taste value (T) between the different PMs (except for H and T) and varieties (Table 4). Additionally, all indicators, except for A and T, demonstrated significant differences under the interaction between PM and variety (Table 4). Compared with DS, TP significantly increased A, Ss, and DB by 2.61%, 7.15%, and 3.19%, respectively. LX21 exhibited significantly higher A, Ss, DB, and T values than H9710 and LY7362, whereas its H value was 4.92% lower than that of the other two varieties. These results suggest that LX21 has superior taste quality compared to H9710 and LY7362.

### 3.4. Nutritional Quality of Rice

The analysis of variance revealed significant differences in AAC%, Fa, Fb_3_, Fa/Fb_3_, and PC% among different PMs (except for PC%) and varieties, whereas only Fa and PC% showed significant differences under the interaction condition (Table 5). Compared with DS, TP significantly increased AAC%, Fa, Fa/Fb_3_, and PC% by 6.20%, 0.66%, 5.34%, and 19.93%, respectively, whereas Fb_3_ decreased by 4.23%. LY7362 exhibited a higher AAC%, Fb_3_, and PC% than LX21 and H9710, whereas the opposite trend was observed for Fa/Fb_3_, which was significantly lower in LY7362. Additionally, H9710 had significantly higher Fa and Fa/Fb_3_ ratios than those of LX21. These results suggest that TP can enhance the nutritional quality of rice, with H9710 demonstrating superior nutritional quality compared to LX21 and LY7362.

### 3.5. RVA of Rice

The analysis of variance revealed significant differences in PV, TV, FV, BD, SB, PeT, and PaT among the different PMs (except for TV, BD, and PeT) and varieties (except for SB). Additionally, all indicators, except for TV and PaT, showed significant differences under the interaction between PM and variety (Table 6). Compared with DS, TP resulted in higher PV, FV, and PaT, while significantly decreasing SB. H9710 exhibited the significantly higher PV, TV, BD, PeT, and PaT than LX21 and LY7362, while LX21 had a significantly greater FV than both H9710 and LY7362. These findings indicated that under TP conditions SB decreased, whereas PV, FV, and PaT increased, with PeT remaining unaffected by PM changes. Additionally, the FV of upland rice variety H9710 was lower than that of paddy rice varieties LX21 and LY7362, whereas SB showed no significant difference among the three varieties. However, all other RVA profile characteristic values of H9710 were higher than those of the paddy rice varieties.

### 3.6. Correlation Between Nutritional Quality and RVA of Rice

**Figure 2:**
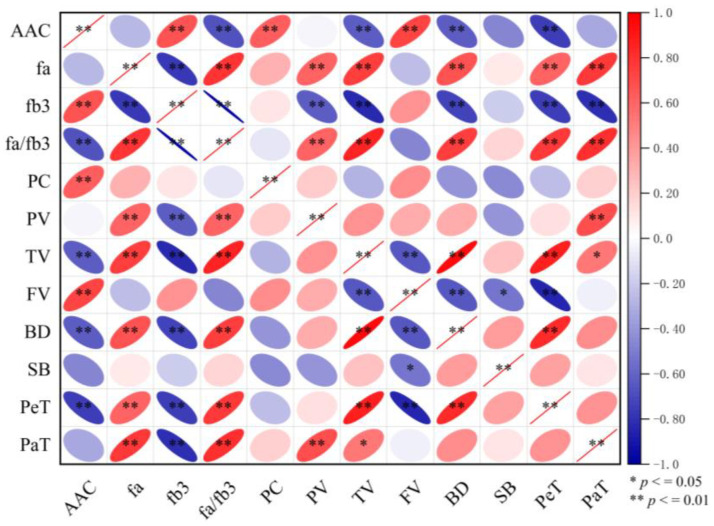
Correlation between physical and chemical properties of rice and food quality. Note: AAC% is short for apparent amylose content; fa stands for short-chain starch; fb_3_ stands for long-branched starch; Fa/Fb_3_ is the ratio of short-chain to long-chain starch; PC% is short for protein content. PV is short for peak viscosity; TV is short for trough viscosity; FV is short for final viscosity; BD is short for breakdown; SB is short for setback; PeT is short for peak time; PaT is short for pasting temperature. *, ** indicate significant differences at *p* ≤ 0.05 and 0.01, respectively.

The results of the correlation analysis (Figure 2) indicated that AAC% had a highly significant positive correlation with Fb_3_, PC%, and FV but a highly significant negative correlation with Fa/Fb_3_, TV, BD, and PeT. Fa exhibited a highly significant positive correlation with Fa/Fb_3_, PV, TV, BD, PeT, and PaT and a highly significant negative correlation with Fb_3_. Fb_3_ levels exhibited a highly significant positive correlation with Fa/Fb_3_, PV, TV, BD, PeT, and PaT. Similarly, the Fa/Fb_3_ ratio was positively correlated with PV, TV, BD, PeT, and PaT. PV showed a highly significant positive correlation with PaT, whereas TV had a significant positive correlation with BD, PeT, and PaT but a highly significant negative correlation with FV. FV displayed a significant negative correlation with BD, SB, and PeT, whereas BD had a highly significant positive correlation with PeT.

### 3.7. Influence of Climatic Factors on Rice Quality and RVA Curves

Studies have suggested that the 20-day grain-filling and seed-setting stages are crucial for rice quality formation, as temperature plays a significant role during this period [20,21]. Therefore, this study recorded the temperature and sunshine factors in August and September for the rice varieties (Table 7). The results indicated that the average air temperatures of the three varieties remained stable under both TP and DS. However, the maximum air temperature, minimum air temperature, sunshine hours, and daily average temperature followed a pattern of first decreasing, then increasing, and finally stabilizing. In addition, these trends appeared earlier under DS than under TP.

Correlation analysis (Table 8) demonstrated that the Fa content had a significant positive correlation with the daily mean temperature and daily lowest temperature but a significant negative correlation with the daily mean light hours. Fb_3_ exhibited a highly significant negative correlation with the daily mean temperature and daily lowest temperature and a highly significant positive correlation with the daily mean temperature difference and daily mean light hours. Fa/Fb_3_ had a highly significant positive correlation with the daily mean temperature and daily lowest temperature but a highly significant negative correlation with the daily mean temperature difference and daily mean light hours. PV was significantly positively correlated with the daily mean temperature, daily highest temperature, and daily lowest temperature, while displaying a highly significant negative correlation with the daily mean temperature difference and daily mean light hours. TV and PeT had significant positive correlations with daily mean temperature. PaT exhibited a highly significant positive correlation with the daily mean temperature, daily highest temperature, and the daily lowest temperature, while also showing a highly significant negative correlation with the daily mean light hours. The remaining quality indicators showed no significant correlation with the measured temperature and light factors.

## 4. Discussion

PMs can directly influence rice growth and substance accumulation at various stages including sowing, vegetative growth, grain filling, seed setting, and maturity. These physiological processes may vary significantly with different PMs, thereby affecting overall growth rhythm, yield performance, and rice quality. Despite the growing importance of planting techniques in rice cultivation, most existing studies have focused on improving cultivation techniques for single varieties, with limited systematic research on quality differences among different rice varieties under varying planting patterns and their impact on the physical and chemical properties of rice. Further exploration of this area is crucial for optimizing rice cultivation management and enhancing its quality [22,23]. This study demonstrated that PMs, japonica rice varieties, and their interactions significantly influenced rice processing, appearance, tasting, and nutritional quality.

### 4.1. Effects of Planting Methods and Varieties on the Processing and Appearance Quality of Rice

The processing quality of rice is largely influenced by the degree of grain filling, which is affected by factors such as photosynthetic efficiency, population structure, temperature, and light. These factors can affect the grain-filling process, and consequently, the processing characteristics and overall quality of rice. Additionally, different cultivation methods also affect processing quality [24,25]. The results of this study indicated that although TP and DS involved different sowing methods, certain environmental factors, such as soil, climate, moisture, and nutrients, remained stable during rice growth and development, leading to relatively small differences in processing quality. For the same variety, TP and DS had minimal and nonsignificant effects on BRR, MRR, and HRR. However, differences were observed in processing quality among the tested varieties. While the BRR remained consistent across varieties, LX21 exhibited a higher MRR and HRR than H9710 and LY7362. This suggested that despite having a similar rice yield rate, LX21 produced less broken rice and had a higher HRR, resulting in greater commercial value.

The primary appearance quality indicators for evaluating rice include the grain type, CH, and CGR. Compared with TP, DS resulted in a slight increase in GL, slight decrease in GW, and significant increase in GL/W, altering the rice shape from short and thick to slender; short and thick rice grains are less prone to breakage during processing [26,27]. High temperatures can accelerate grain filling and growth, causing an uneven distribution of substances within the grains and increasing CH, whereas lower temperatures can help reduce chalky grain formation. Because the temperature during the filling period under DS is lower than that under TP, DS can improve the quality of rice appearance [28]. Bian et al.’s research shows that CGR, CH, and chalky size can be significantly lower under DS conditions than under TP conditions [29]. The results of this study further indicated that, compared to TP, rice varieties under DS conditions showed significantly increased GL and GW, but a significantly reduced GL/W. Furthermore, CGR and CH were significantly lower under DS than under TP, thus demonstrating that the direct seeding method can enhance rice appearance quality. LX21 exhibited significantly higher GL and GL/W and significantly lower CGR and CH than H9710 and LY7362; longer grain length combined with low CGR and CH confer higher market value and appeal. This indicates that LX21 outperforms H9710 and LY7362 in terms of appearance quality. Selecting a suitable japonica rice variety under DS is beneficial for improving the quality of rice appearance, with LX21 being a promising option. Therefore, choosing the appropriate PMs and japonica rice varieties is essential for enhancing the quality of rice appearance.

### 4.2. Effects of Planting Methods and Varieties on the Tasting Quality, Nutritional Quality, and RVA of Rice

The tasting quality of rice can be influenced by the H and Ss of cooked rice [30]. The results of this study presented no significant differences in H and T between the varieties under TP and DS. However, A, Ss, and DB were higher under TP than under DS. Additionally, LX21 exhibited significantly higher A, Ss, DB and T than H9710 and LY7362, indicating that planting patterns had a relatively minor effect on tasting quality, while variety was the key factor influencing tasting quality. Overall, LX21 demonstrated superior taste quality compared with the other varieties, suggesting that selecting an appropriate variety was more effective in improving rice tasting quality.

AAC% and PC% are key indicators of rice nutritional quality, with higher PC% leading to harder rice and poorer palatability when AAC% is similar [31]. Xing et al. reported that rice grown under DS had relatively lower protein content than that grown under TP [32]. This study suggested that PMs had a minimal effect on rice PC%, with no significant differences observed. However, H9710 had a significantly lower PC% than that of LX21 and LY7362. The rice PC% was influenced by multiple factors, including climate and soil, with variety and cultivation practices being the primary determinants. Although different PMs had a relatively minor effect on PC%, variety selection played a more significant role under the same conditions. Therefore, selecting and cultivating suitable rice varieties could be the most effective way to improve the PC%.

Rice with a low AAC% and a long gel consistency exhibits superior cooking and eating qualities [33,34]. DS treatment can result in a higher AAC% and reduce gel consistency, leading to poorer cooking and eating quality [35]. In this study, AAC% and Fa were lower under DS than under TP, suggesting that the comprehensive tasting quality of rice under TP was significantly lower than that under DS. Additionally, H9710 had a significantly higher comprehensive tasting quality value than LX21 and LY7362. The DS method increased GL, produced plumper grains, and had a lower AAC% and higher Ss. Therefore, selecting suitable japonica rice varieties for DS can effectively enhance the cooking and tasting qualities of rice.

The RVA profile of starch is a key indicator of rice cooking and tasting quality [34]. Compared with carpet seedlings, mechanical DS significantly reduces PV and BD while increasing SB [29]. The results of this study demonstrated that PV, FV, and PaT were higher under TP than under DS, whereas SB was lower under TP. No significant differences were observed on TV, BD, or PeT between the TP and DS groups. H9710 exhibited significantly higher PV, TV, BD, PeT, and PaT than LX21 and LY7362, while LX21 had a significantly greater FV than both H9710 and LY7362. These results revealed that under TP, SB decreased, whereas PV, FV, and PaT increased, with TV, BD, and PeT remaining unaffected by the utilized PMs. Additionally, the upland rice variety H9710 had a significantly lower FV than the paddy rice varieties LX21 and LY7362, whereas SB demonstrated no differences among the three varieties. This suggested that both planting methods and rice varieties had a considerable impact on the characteristic values of the rice RVA profile.

### 4.3. Correlation Between Nutritional Quality and RVA of Rice

Rice quality is a complex quantitative trait that is regulated by multiple genes and is influenced by genetic, environmental, and cultivation factors [36]. Rice varieties with a higher tasting quality tend to have a lower AAC% [37]. For PC%, a threshold of 7% can be used to classify the quality with values below 7%, indicating high-quality rice with better taste, whereas values above 7% denote poorer taste with decreasing quality as PC% increases [38]. This study revealed differences in nutritional quality among the varieties, which are consistent with the findings of Chen et al. [39]. The AAC% trend was consistent with the mass fraction of Fb_3_, indicating that a higher AAC% corresponded to a higher Fb_3_, whereas Fa/Fb_3_ was negatively correlated with AAC%. Rice varieties with a high AAC% typically had lower Fa/Fb_3_ values. Additionally, as PC% increases, AAC% tends to decrease, and vice versa [39,40,41]. However, in contrast to previous findings, this study found a significant positive correlation between AAC% and PC%. Although there was no direct correlation between AAC% and PC%, both factors jointly influenced the rice-eating quality and processing characteristics. A higher AAC% can contribute to a firmer cooked rice texture, whereas PC% may also affect the texture and taste to some extent [16,42]. Chen et al. reported no significant correlation between PC% and Fa, Fb_3_, or Fa/Fb_3_, although in some cases, the PC% changes may indirectly influence the amylopectin synthesis and accumulation, thereby affecting Fa, Fb_3_, and Fa/Fb_3_ [39], which can be consistent with the findings of this study.

There can be a correlation among various RVA parameters. Yu et al. observed no direct correlation between PV and PaT, whereas TV exhibited a highly significant positive correlation with FV, BD, PeT, and PaT [43]. FV showed no correlation with BD but had a significant positive correlation with SB and PeT, whereas BD had a highly significant positive correlation with PeT. The results of this study differed slightly, indicating that PV had a highly significant positive correlation with PaT. TV exhibited a significant positive correlation with BD, PeT, and PaT but a highly significant negative correlation with FV. FV was significantly negatively correlated with BD, SB, and PeT, whereas BD had a highly significant positive correlation with PeT. Overall, the RVA values were interrelated, and this correlation was influenced by multiple factors.

The taste analyzer output revealed that milled rice chemical composition is significantly correlated with the RVA profile, and that the main constituents of milled rice are protein and amylose [44]. Chen et al. revealed that AAC% had a significant or highly significant negative correlation with BD and a highly significant positive correlation with FV and SB, while PC% showed no significant correlation with the RVA parameters [39]. BD exhibited a significant positive correlation with Fa/Fb_3_ and a significant or highly significant negative correlation with the mass fraction of Fb_3_. Additionally, FV and SB have a highly significant positive correlation with the mass fraction of Fb_3_ and a highly significant negative correlation with Fa/Fb_3_ [39]. The findings of this study differed slightly, showing that AAC% had a highly significant positive correlation with FV but a highly significant negative correlation with TV, BD, and PeT. Fa exhibited a highly significant positive correlation with PV, TV, BD, PeT, and PaT, whereas Fb_3_ also showed a highly significant positive correlation with these parameters. Overall, there was a clear correlation between RVA profile characteristics and rice nutritional quality, although further research is needed to fully understand these relationships.

### 4.4. Influence of Climatic Factors on Rice Quality

In the same variety, climatic environment is the most critical factor influencing rice quality. The effects of light and temperature on rice quality formation exhibit mutual interactions. For instance, increased light intensity enhances photosynthesis and promotes carbohydrate accumulation, while simultaneously raising temperatures accelerate the grain filling and ripening process, shortening the grain-filling period and thus disadvantaging quality development [45,46,47]. Previous studies have indicated that high temperatures during the grain-filling period can increase CH, reduce grain transparency, GL/W, and HRR, alter the starch structure, expand inter-particle spaces, and significantly impact AAC% [48]. Additionally, high temperatures can elevate SB and PaT in the RVA profile, decrease BD, and extend PeT [36,48]. These changes in crystal structure may be closely linked to variations in amylose content [49,50]. Starch can be fully gelatinized to form a disordered structure under specific moisture and temperature conditions. When gelatinized starch is cooled, the high-energy disordered amylose and amylopectin chains gradually recombine into different ordered structures, eventually crystallizing and reaching a stable ordered state. This process is influenced by multiple factors, including starch composition, other food ingredients, and processing conditions [51,52]. This study found no significant correlations between climatic factors and AAC%, PC%, TV, FV, BD, or SB. However, significant correlations were observed between Fa, Fb_3_, Fa/Fb_3_, PV, TV, PeT, and PaT. Specifically, Fa, Fb_3_, Fa/Fb_3_, PV, and PaT were significantly influenced by fluctuations in daily mean temperature, daily lowest temperature, and daily mean light hours, indicating that temperature and light fluctuations are closely associated with amylopectin accumulation and starch pasting properties.

## 5. Conclusions

In conclusion, PMs had a relatively small impact on processing quality but significantly affected appearance quality, tasting quality, nutritional quality, and RVA profile characteristics. Compared with TP, DS increased GL by 1.19% and GW by 2.69%, reduced CGR by 46.00% and CH by 52.76%, enhanced rice appearance, and increased its commercial value. Additionally, DS resulted in better AAC%, PC%, and FV, leading to significantly higher comprehensive eating quality than TP. Under the same planting conditions, the conventional rice variety LX21 exhibited excellent processing, appearance, and tasting quality, whereas the upland rice variety H9710 exhibited superior nutritional quality and RVA characteristics. Temperature/light variations significantly influence the contents of Fa, Fb_3_, Fa/Fb_3_, PV, and PaT in rice quality. Therefore, selecting appropriate PMs and japonica rice varieties is crucial for improving rice quality. The cultivation method of DS has been widely promoted in countries such as the United States and Australia. This approach not only saves human, material, and environmental resources but also effectively improves the quality of rice cultivars. Therefore, this method should be widely promoted in regions suitable for DS implementation. Enhancing rice quality requires a scientific and well-balanced planting approach, combined with high-quality rice varieties. This not only optimizes the rice growth environment but also improves yield, enhances quality, and supports the sustainable development of the rice industry. As climate change continues, future research will investigate the impacts of microbial variations under different planting conditions on the quality of diverse rice varieties. Meanwhile, biological techniques such as metabolomics will be utilized to unravel the mechanisms underlying rice quality formation.

## Figures and Tables

**Table 1:** The main growth stages of rice under different planting methods (m/d).

PM	V	SS	ES	FHS	MS
TP	LX21	4/15	7/12	8/7	9/29
	H9710	4/15	7/10	8/3	9/26
	LY7362	4/15	7/16	8/15	10/1
DS	LX21	4/27	7/20	8/11	10/1
	H9710	4/27	7/17	8/8	9/30
	LY7362	4/27	7/26	8/20	10/5

Note: PM is short for planting method; V is short for variety; TP is short for transplanting; DS is short for direct seeding; LX21 is short for Liaoxing21; H9710 is short for Han 9710; LY7362 is short for Liaoyou 7362; SS is short for seeding stage; ES is short for elongation stage; FHS is short for full heading stage; MS is short for maturation stage.

**Table 2:** Effects of planting methods on rice grain milling quality of different varieties.

Items	V	BRR (%)	MRR (%)	HRR (%)
TP	LX21	0.81ab	0.71a	68.09a
	H9710	0.81a	0.71a	64.21b
	LY7362	0.81ab	0.68a	60.40d
DS	LX21	0.81ab	0.71a	68.97a
	H9710	0.81ab	0.68a	63.39bc
	LY7362	0.81b	0.69a	61.17cd
	LSD (0.05)	0.01	0.04	2.56
PM				
TP		0.81a	0.70a	64.23a
DS		0.81a	0.69a	64.51a
V				
LX21		0.81ab	0.71a	68.53a
H9710		0.81a	0.69ab	63.80b
LY7362		0.81b	0.68b	60.78c
Analysis of variance				
PM		NS	NS	NS
V		NS	*	**
PM × V		NS	NS	NS

Note: PM is short for planting method; V is short for variety; TP is short for transplanting; DS is short for direct seeding; LX21 is short for Liaoxing21; H9710 is short for Han 9710; LY7362 is short for Liaoyou 7362; BRR is short for brown rice rate; MRR is short for milled rice rate; HRR is short for head rice rate. In the table, a, b, c, d signify significant differences (*p* < 0.05) between the treatments within the same column. *, ** indicate significant differences at *p* ≤ 0.05 and 0.01, respectively.

**Table 3:** Effects of planting methods on rice grain appearance of different varieties.

Items	V	GL (mm)	GW (mm)	GL/W	CGR (%)	CH (%)
TP	LX21	5.80a	2.50d	2.32a	0.85c	1.55c
	H9710	4.70c	2.70b	1.74c	1.59b	3.51a
	LY7362	4.60d	2.60c	1.77b	2.06a	3.65a
DS	LX21	5.80a	2.50d	2.32a	0.23d	0.45d
	H9710	4.80b	2.80a	1.71d	1.37b	2.36b
	LY7362	4.70c	2.70b	1.74c	0.83c	1.30c
	LSD (0.05)	0.02	<0.01	0.01	0.29	0.57
PM						
TP		5.04b	2.60b	1.94a	1.50a	2.90a
DS		5.10a	2.67a	1.92b	0.81b	1.37b
V						
LX21		5.80a	2.50c	2.32a	0.54b	1.00c
H9710		4.75b	2.75a	1.73c	1.48a	2.94a
LY7362		4.65c	2.65b	1.75b	1.45a	2.48b
Analysis of variance						
PM		**	**	**	**	**
V		**	**	**	**	**
PM × V		**	**	**	**	**

Note: PM is short for planting method; V is short for variety; TP is short for transplanting; DS is short for direct seeding; LX21 is short for Liaoxing21; H9710 is short for Han 9710; LY7362 is short for Liaoyou 7362; GL is short for grain length; GW is short for grain width; GL/W is short for grain length/width; MS is short for maturation stage, CGR is short for chalkiness grain rate; CH is short for chalkiness. In the table, a, b, c, d signify significant differences (*p* < 0.05) between the treatments within the same column. **, indicate significant differences at *p* ≤ 0.01.

**Table 4:** Effects of planting methods on rice grain taste.

Items	V	A	H	Ss	DB	T
TP	LX21	7.03a	6.03d	7.03a	7.07a	71.67a
	H9710	6.43c	6.67a	6.73c	6.53cd	67.67b
	LY7362	6.60b	6.47b	6.90ab	6.80b	70.33ab
DS	LX21	6.93a	6.33c	6.77bc	6.63bc	70.33ab
	H9710	6.30c	6.33c	6.00e	6.70bc	68.00b
	LY7362	6.33c	6.53b	6.53d	6.43d	69.00ab
	LSD (0.05)	0.16	0.11	0.15	0.19	3.08
PM						
TP		6.69a	6.39a	6.89a	6.80a	69.89a
DS		6.52b	6.40a	6.43b	6.59b	69.11a
V						
LX21		6.98a	6.18b	6.90a	6.85a	71.00a
H9710		6.37b	6.50a	6.37c	6.62b	67.83c
LY7362		6.47b	6.50a	6.72b	6.62b	69.67b
Analysis of variance						
PM		*	NS	**	*	NS
V		**	**	**	**	**
PM × V		NS	**	**	**	NS

Note: PM is short for planting method; V is short for variety; LX21 is short for Liaoxing21; H9710 is short for Han 9710; LY7362 is short for Liaoyou 7362; A is short for appearance; H is short for hardness; Ss is short for stickiness; DB is short for degree of balance; T is short for taste value. In the table, a, b, c, d, e signify significant differences (*p* < 0.05) between the treatments within the same column. *, ** indicate significant differences at *p* ≤ 0.05 and 0.01, respectively.

**Table 5:** Effects of planting methods on rice nutrition of different varieties.

Items	V	AAC%	fa	fb_3_	fa/fb_3_	PC%
TP	LX21	19.10ab	31.95ab	6.39b	5.00c	7.67a
	H9710	17.22d	32.14a	5.06d	6.35a	7.27abc
	LY7362	19.67a	31.85b	6.86a	4.65d	7.43ab
DS	LX21	17.58cd	31.48c	6.74a	4.68d	6.20bc
	H9710	16.74d	32.08a	5.39c	5.95b	6.04c
	LY7362	18.40bc	31.76b	7.00a	4.54d	6.90abc
	LSD (0.05)	0.91	0.20	0.26	0.20	1.26
PM						
TP		18.66a	31.98a	6.11b	5.33a	7.46a
DS		17.57b	31.77b	6.38a	5.06b	6.38a
V						
LX21		18.34b	31.71b	6.57b	4.84b	6.94ab
H9710		16.98c	32.11a	5.23c	6.15a	6.66b
LY7362		19.04a	31.80b	6.93a	4.59c	7.17a
Analysis of variance						
PM		*	*	*	*	NS
V		**	**	**	**	*
PM × V		NS	**	NS	NS	*

Note: PM is short for planting method; V is short for variety; TP is short for transplanting; DS is short for direct seeding; LX21 is short for Liaoxing21; H9710 is short for Han 9710; LY7362 is short for Liaoyou 7362; AAC% is short for apparent amylose content; fa stands for short-chain starch; fb3 stands for long-branched starch; Fa/Fb3 is the ratio of short-chain to long-chain starch; PC% is short for protein content. In the table, a, b, c and d signify significant differences (*p* < 0.05) between the treatments within the same column. *, ** indicate significant differences at *p* ≤ 0.05 and 0.01, respectively.

**Table 6:** Effects of planting methods on RVA characteristics of rice varieties.

Items	V	PV/cP	TV/cP	FV/cP	BD/cP	SB/cP	PeT (min)	PaT/°C
TP	LX21	3572.67a	1585.00bc	1870.33a	3142.67d	1607.67ab	5.67c	71.02a
	H9710	3542.67ab	2058.33a	1424.33c	3538.33b	1580.00b	6.13a	71.77a
	LY7362	3495.33ab	1668.33bc	1841.00a	3235.33cd	1500.33c	5.69c	69.63b
DS	LX21	3409.67b	1506.00c	1650.00b	3089.67d	1596.67ab	5.71c	69.37b
	H9710	3124.00c	2151.33a	1427.33c	3801.00a	1649.67a	6.11a	71.02a
	LY7362	2800.00d	1724.33b	1375.67c	3337.33c	1646.33ab	5.93b	68.85b
	LSD (0.05)	136.98	203.39	56.89	167.04	68.73	0.17	1.22
PM								
TP		3508.33a	1770.56a	1711.89a	3305.44a	1562.67b	5.83a	70.81a
DS		3139.78b	1793.89a	1484.33b	3409.33a	1630.89a	5.92a	69.74b
V								
LX21		3266.83b	1545.5c	1760.17a	3116.17c	1602.17a	5.69c	70.19b
H9710		3534.00a	2104.83a	1425.83c	3669.67a	1614.83a	6.12a	71.39a
LY7362		3171.33c	1696.33b	1608.33b	3286.33b	1573.33a	5.81b	69.24c
Analysis of variance								
PM		**	NS	**	NS	*	NS	*
V		**	**	**	**	NS	**	**
PM × V		**	NS	**	*	*	**	NS

Note: PM is short for planting method; V is short for variety; TP is short for transplanting; DS is short for direct seeding; LX21 is short for Liaoxing21; H9710 is short for Han 9710; LY7362 is short for Liaoyou 7362; PV is short for peak viscosity; TV is short for trough viscosity; FV is short for Final viscosity; BD is short for breakdown; SB is short for setback; PeT is short for peak time; PaT is short for pasting temperature. In the table, a, b, c, d signify significant differences (*p* < 0.05) between the treatments within the same column. *, ** indicate significant differences at *p* ≤ 0.05 and 0.01, respectively.

**Table 7:** Differences in temperature and sunshine factors in the grain-filling and seed-setting period of rice varieties.

Tems	V	Daily Mean Temperature (°C)	Daily Highest Temperature (°C)	Daily Lowest Temperature (°C)	Daily Mean Temperature Difference (°C)	Daily Mean Light Hours (h)
TP	LX21	21.18	26.63	15.73	10.90	6.44
	H9710	21.61	26.87	16.35	10.51	6.02
	LY7362	20.09	26.48	14.63	11.54	7.12
DS	LX21	20.37	26.42	15.19	10.95	6.66
	H9710	21.07	26.58	15.55	11.03	6.59
	LY7362	20.20	25.70	13.44	12.13	7.54

Note: V is short for variety; TP is short for transplanting; DS is short for direct seeding; LX21 is short for Liaoxing21; H9710 is short for Han 9710; LY7362 is short for Liaoyou 7362.

**Table 8:** Correlation analysis of rice quality and RVA with temperature and light factors during the grain-filling and seed-setting period of rice varieties.

Rice Quality Character	Daily Mean Temperature (°C)	Daily Highest Temperature (°C)	Daily Lowest Temperature (°C)	Daily Mean Temperature Difference (°C)	Daily Mean Light Hours (h)
AAC%	−0.44	−0.18	−0.40	0.41	0.44
fa	0.63 **	0.34	0.50 *	−0.35	−0.47 *
fb_3_	−0.75 **	−0.45	−0.75 **	0.69 **	0.77 **
fa/fb_3_	0.75 **	0.44	0.73 **	−0.67 **	−0.75 **
PC%	0.09	0.06	0.05	−0.01	−0.07
PV/cP	0.49 *	0.63 **	0.75 **	−0.63 **	−0.66 **
TV/cP	0.52 *	0.24	0.37	−0.26	−0.36
FV/cP	−0.17	0.21	0.12	−0.10	−0.03
BD/cP	0.44	0.19	0.26	−0.13	−0.22
SB/cP	0.29	−0.06	−0.04	0.12	0.06
PeT (min)	0.50 *	0.14	0.26	−0.16	−0.27
PaT/°C	0.81 **	0.60 **	0.82 **	−0.69**	−0.78 **

Note: AAC% is short for apparent amylose content; fa stands for short-chain starch; fb stands for long-branched starch; Fa/Fb_3_ is the ratio of short-chain to long-chain starch; PC% is short for protein content. PV is short for peak viscosity; TV is short for trough viscosity; FV is short for final viscosity; BD is short for breakdown; SB is short for setback; PeT is short for peak time; PaT is short for pasting temperature. *, ** indicate significant differences at *p* ≤ 0.05 and 0.01, respectively.

## Data Availability

The original contributions presented in this study are included in the article. Further inquiries can be directed to the corresponding authors.

## Abbreviations

The following abbreviations are used in this manuscript: PM is planting method; TP is transplanting; DS is direct seeding; V is variety; LX21 is Liaoxing21; H9710 is Han 9710; LY7362 is Liaoyou 7362; SS is seeding stage; ES is elongation stage; FHS is full heading stage; MS is maturation stage; BRR is brown rice rate; milled rice rate is MRR; head rice rate is HRR; GL is grain length; GW is grain width; GL/W is grain length/width; MS is maturation stage, CGR is chalkiness grain rate; CH is chalkiness; AAC% is apparent amylose content; PC% is protein content; PV is peak viscosity; TV is trough viscosity; FV is final viscosity; BD is breakdown; SB is setback; PeT is peak time; and PaT is pasting temperature.
